# Clinical Review of Microbial Corneal Ulcers Resulting in Enucleation and Evisceration in a Tertiary Eye Care Center in Hungary

**DOI:** 10.1155/2020/8283131

**Published:** 2020-05-18

**Authors:** Gábor Tóth, Milán Tamás Pluzsik, Gábor László Sándor, Orsolya Németh, Olga Lukáts, Zoltán Zsolt Nagy, Nóra Szentmáry

**Affiliations:** ^1^Department of Ophthalmology, Semmelweis University, Budapest, Hungary; ^2^Department of Ophthalmology, Bajcsy-Zsilinszky Hospital, Budapest, Hungary; ^3^Department of Ophthalmology, Saarland University Medical Center (UKS), Homburg, Saar, Germany

## Abstract

**Purpose:**

To analyse the clinical and microbiological characteristics and preexisting ophthalmic and systemic conditions of infectious keratitis resulting in enucleation/evisceration in a large tertiary referral center in a developed country (Hungary) over a period of 12 years. *Patients and Methods*. A retrospective review of enucleated/eviscerated eyes undergoing surgery between 2007 and 2018 at the Department of Ophthalmology of Semmelweis University, Budapest, Hungary, with infectious keratitis as the primary indication for enucleation or evisceration. For each subject, clinical history, B-scan ultrasound report, and microbiological analyses were reviewed.

**Results:**

There were 48 enucleated/eviscerated eyes from 47 patients (29 females (61.7%), age at the time of surgery 66.4 ± 18.5 years). Indication for surgery was hopeless, unmanageable keratitis (62.5%), and keratitis with endophthalmitis (37.5%). The most common preexisting ophthalmic conditions were previous cataract surgery (60.4%), previous therapeutic penetrating keratoplasty (PKP) (56.3%), corneal perforation (52.1%), glaucoma (41.7%), and long-term topical steroid usage (31.3%). In order to treat keratitis, before enucleation or evisceration, 20 eyes (41.7%) underwent PKP, 12 eyes (25.0%) amniotic membrane transplantation, 8 eyes (16.7%) conjunctival autograft transplantation, 6 eyes (12.5%) tarsorrhaphy, and 4 eyes (8.3%) vitrectomy to salvage the eye prior to the final treatment of enucleation or evisceration. The most frequent preexisting systemic diseases were hypertension (62.5%), cardiac disease (20.8%), diabetes mellitus (20.8%), and rheumatoid arthritis (14.6%). *Staphylococcus aureus* (17.0%) and *Propionibacterium acnes* (12.8%) were the most commonly isolated gram-positive bacteria, and *Pseudomonas aeruginosa* was the most frequently isolated gram-negative pathogen bacterium (10.6%). Six globes (12.5%) had positive fungal cultures (1 case of *Candida albicans*, *Candida parapsilosis*, *Trichosporon inkin*, *Acremonium* sp., *Fusarium* sp., and *Penicillium* sp.).

**Conclusions:**

*Staphylococcus aureus, Propionibacterium acnes*, and *Pseudomonas aeruginosa* keratitis with or without endophthalmitis represent the most common indication for ocular enucleation/evisceration in patients with microbial keratitis in a tertiary referral center in Hungary. The incidence of enucleation and evisceration related to mycotic keratitis does not seem to have increased within the last decade. Most frequent preexisting systemic diseases in cases of enucleation and evisceration are hypertension, cardiac disease, diabetes mellitus, and rheumatoid arthritis.

## 1. Introduction

Infectious keratitis is the leading cause of unilateral blindness worldwide [1]. Enucleation and evisceration are sometimes unavoidable end-stage solutions for several ophthalmic diseases. These may be required due to infection (commonly in Asia and Africa), after severe ocular trauma, in cases of tumour or painful blind eye [2].

Basically, enucleation and evisceration should be avoided in infections involving only the anterior segment [3]. Nevertheless, these are indicated in cases where microbial keratitis progresses despite maximal appropriate medical and surgical treatment and the entire globe is compromised.

The incidence of different microorganisms and their resistance pattern varies worldwide and changes over time. Therefore, ophthalmologists also see microorganisms that lead to severe keratitis and loss of the eye and have to analyse the changing trends of microbial resistance from time to time [4].

Several previous studies have analysed corneal ulcers/keratitis that have resulted in enucleation or evisceration in different countries [5–7], but there is no current information available for European countries and in the continental climate.

The primary aim of this study was to analyse current clinical and microbial indications for enucleation and evisceration in infectious keratitis at a tertiary eye care center in a European developed country (Hungary) over a period of 12 years.

## 2. Materials and Methods

This retrospective study was undertaken at a tertiary eye care center, to analyse the current clinical and microbial indications of enucleation and evisceration in infectious keratitis in Hungary. The study was approved by the Regional and Institutional Committee of Science and Research Ethics of Semmelweis University, Hungary (Number 122/2019). The study was performed in accordance with the Declaration of Helsinki Guidelines for Human Research.

In total, 583 eyes of 579 patients underwent enucleation or evisceration over a period of 12 years (between January 2007 and December 2018) at the Department of Ophthalmology of Semmelweis University. Among these, 48 eyes of 47 patients were removed due to microbial keratitis. Our study was conducted on these 48 eyes.

The diagnosis of “infectious keratitis” was made based on the clinical findings and characteristics and was defined as the presence of corneal infiltrate [5]. Corneal ulcer was considered to be central if it affected the central one-third, midperipheral if it affected the middle one-third, and peripheral if it affected the peripheral one-third of the corneal radius. For each subject, the clinical data were reviewed including patient demographics, clinical history, presenting visual acuity, indication for enucleation and evisceration, microbiological analyses (culture and antibiotic sensitivities), B-scan ultrasound reports, operative details, and results of the histopathological analysis following enucleation or evisceration. Visual acuity data were converted from Snellen chart values to LogMAR format.

Corneal scrapes were obtained with a sterile needle or a hockey knife for Gram's and Grocott stains. Corneal smears were obtained with a swab, a sterile needle or a hockey knife for in vitro culture. In vitro culture included a brain heart infusion broth, thioglycollate broth, chocolate agar, Schaedler anaerobe agar, Columbia colistin-nalidixic agar, and Sabouraud's dextrose agar plates.

Local broad-spectrum antibiotics (levofloxacin 5 mg/ml, moxifloxacin 0.5% or fortified cefazolin sodium 5% and tobramycin sulfate 1.3%), cycloplegic eye drops, and systemic ciprofloxacin 500 mg twice daily were ordered for every patient on first presentation at our clinic. Treatment was then modified if indicated by culture results, antibiotic sensitivity, and clinical response.

## 3. Results

**Figure 1:**
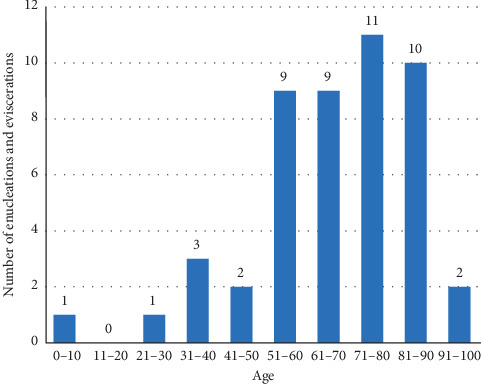
Age distribution (at the time of surgery) of patients who underwent enucleation or evisceration due to infectious keratitis between January 2007 and December 2018 at the Department of Ophthalmology of Semmelweis University (Budapest, Hungary) (48 eyes of 47 patients).

**Figure 2:**
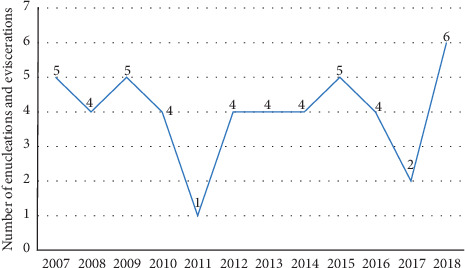
Annual number of enucleations and eviscerations due to microbial keratitis between 2007 and 2018 at the Department of Ophthalmology of Semmelweis University (Budapest, Hungary).

The demographic characteristics and age distribution of the subjects are shown in Table 1 and Figure 1. Medical characteristics of the patients are displayed at Table 2. Annual number of enucleations and eviscerations due to microbial keratitis between 2007 and 2018 is shown at Figure 2.

Indications for surgery were hopeless, unmanageable keratitis in 30 (62.5%) and keratitis with endophthalmitis in 18 (37.5%) eyes. B-scan ultrasound examinations showed endophthalmitis in 18 (37.5%), normal posterior segment in 9 (18.8%), retinal detachment in 9 (18.8%), and choroidal detachment in 5 (10.4%) eyes and was not available in 7 (14.6%) cases.

Associated preexisting ophthalmological factors were found in 45 (93.8%) eyes and preexisting systemic diseases in 41 (85.4%) cases.

The most frequently found preexisting ophthalmological factors (Table 3) at the time of the enucleation or evisceration included previous cataract surgery (*n* = 29; 60.4%), previous corneal transplantation (*n* = 27; 56.3%), corneal perforation (*n* = 25; 52.1%), glaucoma (*n* = 20; 41.7%), previous amniotic membrane transplantation (*n* = 16; 33.3%), and long-term usage of steroid eye drops (*n* = 15; 31.3%). Besides, 3 patients (6.3%) had a positive clinical history of herpetic keratitis in the affected eye.

No patient had a history of a routine ocular surgery in the recent past. In order to manage keratitis, before enucleation or evisceration, 20 eyes (41.7%) underwent PKP, 12 eyes (25.0%) amniotic membrane transplantation, 8 eyes (16.7%) conjunctival autograft transplantation, 6 eyes (12.5%) tarsorrhaphy, and 4 eyes (8.3%) vitrectomy to salvage the eye prior to the final treatment of enucleation or evisceration.

The most commonly associated preexisting systemic diseases (Table 3) at the time of the enucleation and evisceration included hypertension (*n* = 30; 62.5%), cardiac disease (*n* = 10; 20.8%), diabetes mellitus (*n* = 10; 20.8%), and rheumatoid arthritis (*n* = 7; 14.6%). The only patient aged under 18 years had Lyell's syndrome as an associated preexisting systemic disease.

On first presentation at our clinic, 11 patients (22.9%) were using tobramycin 0.3%, 11 (22.9%) levofloxacin 5 mg/ml, 7 (14.6%) ofloxacin 0.3%, and 6 (12.5%) moxifloxacin 0.5% eye drops, and 13 patients (27.1%) were without current topical antibiotic therapy.

Altogether, 37 (77.1%) microbiological cultures were positive.

Microbiological cultures were positive in 34 (70.8%) cases for bacteria, among these 23 (67.6%) were positive for 1, 10 (29.4%) for 2, and 1 (2.9%) for 3 bacterial isolates.

Coagulase negative *Staphylococci* (CoNS) (*n* = 11; 31.4%), *Staphylococcus aureus* (*S. aureus*) (*n* = 8; 22.9%), *Streptococci* (*n* = 7; 20.0%), and *Propionibacterium acnes* (*P. acnes*) (*n* = 6; 17.1%) were the most frequently isolated gram-positive bacteria (Table 4). *Pseudomonas aeruginosa* (*Pseudomonas*) was the most commonly isolated gram-negative bacterium (*n* = 5; 41.7%).

Six eyes (12.5%) had a positive fungal culture (1 case of *Candida albicans*, *Candida parapsilosis*, *Trichosporon inkin*, *Acremonium* sp., *Fusarium* sp., and *Penicillium* sp.). The sample with *Trichosporon inkin* was also culture positive for *Enterobacter cloacae*, the sample with *Candida parapsilosis* was also culture positive for *P. acnes*, and the sample with *Penicillium* sp. was also culture positive for *Streptococcus pneumoniae.*

All of the isolated bacteria were tested for antibiotic susceptibility (Table 5).

Of the tested strains, 50.0% (*n* = 9) were resistant to cefazolin, 31.8% (*n* = 7) to moxifloxacin, 25.0% (*n* = 2) to neomycin, 25.0% (*n* = 7) to tobramycin, 23.3% (*n* = 7) to gentamycin, 21.4% (*n* = 6) to levofloxacin, 16.7% (*n* = 1) to ofloxacin, and 7.7% (*n* = 1) to ceftriaxone. None were resistant to vancomycin.

Antifungal susceptibility for all the isolated fungi is summarized in Table 6. One case of fungal corneal ulcer appeared in 2007, 1 in 2015, 2 in 2017, and 2 in 2018.

Paraffin sections of enucleated and eviscerated globes were stained with hematoxylin-eosin, periodic acid Schiff (PAS), and Gram and Grocott methenamine silver (GMS). Histopathological charts were available for 47 (97.9%) globes. Histopathological examination showed gram-positive cocci in 6 (12.7%), gram-negative rods in 1 (2.1%), and mycotic filaments in 3 (6.4%) cases. Of these 3 samples, microbiological cultures showed *Fusarium* in 1 case and *Trichosporon inkin* in 1 case, and 1 sample was culture negative for fungi. Histopathological examination showed *Acanthamoeba* in 1 (2.1%) case.

In cases with endophthalmitis, the most common isolated microorganisms were *Streptococci* (*n* = 4; 18.2%), fungi (*n* = 4; 18.2%), *Pseudomonas* (*n* = 3; 13.6%), CoNS (*n* = 3; 13.6%), *S. aureus* (*n* = 3; 13.6%), *Acinetobacter baumannii* (*n* = 2; 9.1%), *Proteus mirabilis* (*n* = 1; 4.5%), and *Peptostreptococcus* (*n* = 1; 4.5%). Besides, histopathological examination showed *Acanthamoeba* in 1 case of endophthalmitis. Five patients with endophthalmitis (27.8%) were using steroid eye drops in the long-term.

The only case, which underwent enucleation on both eyes, was a 79-year-old female patient with severe rheumatoid arthritis. She was referred to our clinic with total corneal melting, endophthalmitis, and no light perception on the left eye in 2015 and in the right eye in 2018.

Microbiological analysis was negative in case of the left eye and verified presence of *S. aureus and Streptococcus pyogenes* in case of the right eye.

## 4. Discussion

We analysed the clinical details of microbial keratitis cases that led to severe infectious keratitis and loss of the eye at a tertiary eye care center in Hungary. To the best of our knowledge, this is the first study to report on the properties of infectious keratitis leading to enucleation and evisceration in Europe.

The incidence of infectious keratitis leading to endophthalmitis has been reported to be 6% and leading to enucleation and evisceration to be 1.8% among patients with microbial keratitis in the USA [6, 8]. Keratitis was the primary indication for surgery in 9.0% of all the performed enucleations in Hungary [9]. The most common clinical immediate indications for enucleation and evisceration were nonresolving keratitis in 62.5% and severe keratitis with endophthalmitis in 37.5% of our sample. This is in contrast to Australian data [5] where only 4.3% of the patients had an associated endophthalmitis.

Enucleation and evisceration were performed most frequently in patients between 40 and 92 years of age (91.7%), and the incidence of enucleation and evisceration increased with age in the different age groups. It is known that increased age is one of the main risk factors for infectious keratitis related endophthalmitis and loss of the eye worldwide [10]. Our mean age (66.4 years) was similar as that reported in the USA (67 years) [6], but slightly higher than that recently reported in Thailand (56.5 years) [7].

Associated preexisting systemic and ocular surface diseases, previous corneal surgeries, a positive clinical history of herpetic keratitis, older age, delayed ophthalmological attendance, and previous use of steroid eye drops are all predisposing factors of severe microbial keratitis [11]. In our sample, similar to Hongyok and Leelaprute's [7] and Constantinou et al.'s [5] data, hypertension (62.5% vs. 17% in Thailand), rheumatoid arthritis (14.6% vs. 36% in Australia), and diabetes mellitus (20.8% vs. 13–19%) were the most commonly associated preexisting systemic diseases. High prevalence of hypertension and cardiac diseases is rather considered to be a coincidence with infectious keratitis in our sample, as both hypertension and cardiac disease affect mainly elderly (prevalence of hypertension was estimated to be more than 60% in Hungary in 2017, among people aged 60 years or older) [12]. Several studies have shown that diabetic keratopathy [13] and rheumatoid arthritis related dry eye increase the risk of infectious keratitis [5]. Lyell's syndrome, atopic dermatitis, Sjögren syndrome, and rosacea enhance the vulnerability of the ocular surface and the cornea [14]. Dementia, depression, intellectual disability, alcoholism, noncompliance, and other diseases with cognitive impairment, as well as social isolation in older age, may also make it difficult to follow the instructions of physician and may cause inadequate healing [15]. It is important to remark that almost every fourth patient in our sample had an important associated psychiatric disorder or social problem. If there had been a lack of these differences or a better social safety net these globes could have potentially had a higher chance of salvage.

Similar to Hongyok and Leelaprute's (6%) [7] and Constantinou et al.'s (49%) [5] results, glaucoma was one of the most frequently found associated preexisting eye diseases in our sample (41.7%). In addition, previous cataract (60.4%) and corneal (56.3%) surgery, amniotic membrane (33.3%) and conjunctival autograft (16.7%) transplantation and globe perforation (52.1%), and steroid eye drop usage (31.3%) were the most commonly found associated preexisting ocular factors. Ocular surface diseases, corneal perforation, and previous ocular surgery are well-known risk factors for infectious keratitis associated endophthalmitis [10]. Corneal epithelial defects due to high intraocular pressure and toxic epitheliopathy caused by benzalkonium chloride in topical glaucoma medications, as well as persistent epithelial erosions and corneal innervation changes after corneal transplantation, can increase the risk of infectious keratitis [16, 17]. There was no infectious corneal ulcer less than 3 months after ophthalmic surgery; thus, no early postoperative complications appeared in our sample. Long-term steroid eye drop usage is also a known major risk factor for microbial keratitis and consequent loss of the eye. Topical steroid usage was found to be the most frequently associated risk factor for infectious keratitis induced endophthalmitis in Australia. The incidence of microbial keratitis associated endophthalmitis with the usage of topical steroids was found to be lower in our study when compared to earlier studies (27.8% vs. 37.8–76%) [11].

Generally, ophthalmic patients are treated by general practitioners rather than in primary eye care centers and public hospitals in Hungary. Therapeutic unmanageable cases are referred to tertiary eye care centers. This was also the case for patients of our study. This means that primary treatment, as empiric treatment, was prescribed by general practitioners or general ophthalmologists in most of these cases.

The average duration of symptoms before first examination at our clinic (21.3 days) was similar as that reported in Australia (17.7 days) [5]. In total, 22.9% of our patients used only tobramycin eye drops at the time of first presentation to our clinic. Besides, there was in more than one-third of the cases total corneal melting at this time-point. It is known that prompt diagnosis of microbial keratitis and the early introduction of topical fluoroquinolone or fortified eye drops are essential in the treatment of infectious keratitis [5]. In all probability more eyes might have been saved in our clinic from enucleation and evisceration with the early and aggressive introduction of broad-spectrum antibiotics, particularly as 25.0% of the isolated bacteria were resistant to tobramycin. A few-hour delay in the introduction of therapy may be even more dangerous in cases of severe infectious corneal ulcers [10].

Similar to Manikandan et al. (20.9%) [18] in India, CoNS were the most frequently isolated bacteria (23.4%) along with other gram-positive bacteria (31.4%) from our corneal smears. However, CoNS are also isolated in large numbers from healthy ocular surfaces, as they are a major component of the normal flora of human skin and conjunctiva. Thus, they are considered mostly to be contaminants, to have a low virulence factor and typically do not invade the deeper layers of the cornea [19].

The second most commonly found bacterium was *S. aureus* (17.0%), which is also part of the conjunctival common flora but is also one of the most important pathogens in infectious keratitis and accounts for 8–22% of all bacterial keratitis [20]. In Ong et al.'s report [20] from Taiwan, 8.5% of all patients with therapy refractory *S. aureus* keratitis underwent PKPs, but did not require to perform enucleation or evisceration in the studied 5-year period. Despite the emergence of *methicillin-resistant S. aureus* (MRSA) strains in the world, the prevalence of MRSA was lower (25.0%) in our sample when compared to the latter report from Taiwan (44.0%).

Similar to Constantinou et al.'s report from Australia (8.9%) [5], another commonly detected gram-positive bacterium was *P. acnes* (12.8%). The importance of *P. acnes* is commonly underestimated, as it is considered a commensal bacterium. However, *P. acnes* was reported as a causative factor for 9.3–11.9% of microbial keratitis cases, and even as a causative factor of vision-threatening infectious corneal ulcers [21].

Similar to Constantinou et al.'s study [5], O'Neill et al.'s [11] data from Australia, and Cruzs et al.'s [6] report from the USA, we also found that *Streptococci* (*Streptococcus pneumoniae* and other *Streptococci*) were important gram-positive causative factors of infectious keratitis that resulted in loss of the eye.

Different studies agree that *Pseudomonas*—a gram-negative bacterium—is considered to be the most aggressive bacterium, with a high virulence, which can lead to vision-threatening infectious keratitis, endophthalmitis, and occasionally loss of the eye [5–7]. The incidence of *Pseudomonas* was lower (10.6%) among the isolated bacteria of our sample as compared to Constantinou et al.'s (35%) [5] and Hongyok and Leelaprute's (27.8%) [7] results. O'Neill et al. [11] reported from Australia that 24.3% of all of the microbial keratitis associated endophthalmitis cases were enucleated or eviscerated due to *Pseudomonas* infections, which was higher when compared to our sample (16.7%). *Pseudomonas* can cause complete corneal melting within a short period of time, even within a few hours or days. That is why prompt diagnosis and early aggressive treatment is essential in cases of infectious keratitis caused by *Pseudomonas* [11].

Resistances to tobramycin (25.0% vs. 0%) and ofloxacin (16.7% vs. 0%) were higher in our study when compared to the data of Constantinou et al. [5] from Australia, which may be explained by increasing resistance against these antibiotics between 1998–2007 and 2007–2018. However, some other studies have not reported on increasing resistance against these agents [22]. Systemic ciprofloxacin was given to all patients in our sample at first presentation at our clinic. This fact is noteworthy, as 32.1% of the isolated bacteria were ciprofloxacin resistant. Effect of oral antibiotic use in endophthalmitis treatment is debated. Ciprofloxacin is a second-generation fluoroquinolone with proper ocular penetration and low side effect profile, but resistance against ciprofloxacin has been increasing in the last decades [23, 24]. For this reason, many institutions use moxifloxacin instead of ciprofloxacin for gram-negative bacteria and additionally systemic cefuroxime for gram-positive bacteria [24, 25].

Limitations of our study include the following: It was performed in a retrospective fashion; our department is a tertiary eye care center, and for this reason we could not examine the complete course and progression of these cases and many patients were only referred to our clinic in the terminal phase; treatment regimens varied among ophthalmologists.

## 5. Conclusions

In summary, infectious keratitis caused by *Staphylococcus aureus*, *Streptococci*, *Propionibacterium acnes*, and *Pseudomonas aeruginosa* keratitis with or without endophthalmitis represent the most common indication for ocular enucleation and evisceration in patients with microbial ulcers in Hungary. The incidence of enucleation and evisceration related to mycotic keratitis does not seem to have increased within the last decade. Enucleation and evisceration were performed probably in early phases in some cases, as social indications played an important role in our sample. General practitioners who are often the first point of care should be periodically trained in order to early recognize infectious keratitis and to manage that suitable to the changing microbiological profile. In order to avoid the need for performing enucleation or evisceration, treatment protocols should be standardized, social safety net should be improved in the country, and patients with severe bacterial keratitis should be treated more aggressively in time, based on the knowledge on changing trends in infectious keratitis treatment, because every eye deserves a fair chance.

## Figures and Tables

**Table 1:** Demographic characteristics of patients who underwent enucleation or evisceration due to infectious keratitis between January 2007 and December 2018 at the Department of Ophthalmology of Semmelweis University (Budapest, Hungary) (48 eyes of 47 patients).

Number of patients (*n*)	47
Number of enucleations and eviscerations (*n*)	48
Number of enucleations (*n*)	46 (95.8%)
Number of eviscerations (*n*)	2 (4.2%)
Sex	
Male (*n*)	18 (38.3%)
Female (*n*)	29 (61.7%)
Age at the time of the enucleation or evisceration	66.4 ± 18.5 years
Laterality	
Right (*n*, %)	23 (47.9%)
Left (*n*, %)	25 (52.1%)

**Table 2:** Medical characteristics of patients who underwent enucleation or evisceration due to infectious keratitis between January 2007 and December 2018 at the Department of Ophthalmology of Semmelweis University (Budapest, Hungary) (48 eyes of 47 patients).

Best corrected distance visual acuity (LogMAR)	2.73 ± 0.36
Number of preexisting ocular factors	4 ± 2
Number of preexisting systemic diseases	2 ± 2
Duration of symptoms prior to presentation at our clinic (days)	21.3 ± 34.2
Location of the corneal ulcer	
Central (*n*, %)	18 (37.5%)
Midperipheral (*n*, %)	8 (16.7%)
Peripheral (*n*, %)	5 (10.4%)
Total corneal melting (*n*, %)	17 (35.4%)
Anterior chamber status	
Tyndallization (*n*, %)	8 (16.7%)
Hypopyon (*n*, %)	10 (20.8%)
Loss of anterior chamber (*n*, %)	4 (8.3%)
Not visible or not described (*n*, %)	26 (54.2%)
Average number of attempted surgeries before enucleation or evisceration to manage corneal ulcer	
Penetrating keratoplasty	2 ± 1
Amniotic membrane transplantation	2 ± 1

**Table 3:** Associated preexisting ophthalmological factors and systemic diseases for patients who underwent enucleation or evisceration due to infectious keratitis between January 2007 and December 2018 at the Department of Ophthalmology of Semmelweis University (Budapest, Hungary) (48 eyes of 47 patients).

Ophthalmological factors	*N*	Systemic diseases	*n*
Previous cataract surgery	29	Hypertension	30
Previous corneal transplantation	27	Cardiac disease	10
Corneal perforation	25	Diabetes mellitus	10
Glaucoma	20	Rheumatoid arthritis	7
Amniotic membrane transplantation	16	Previous tuberculosis	3
Long-term topical steroid usage	15	Hypo/hyperthyroidism	3
Conjunctival autograft transplantation	8	Dementia	4
Tarsorrhaphy	7	History of pulmonary embolism	3
Previous vitrectomy	6	Previous stroke	2
Previous ocular trauma	3	Lyell's syndrome	2
Previous herpetic keratitis	3	Depression	2
Uveitis	3	History of vein thrombosis	2
Previous corneal refractive surgery	2	Sjögren's syndrome	2
Previous eyelid tumour	2	Asthma	1
Previous dacryocystitis	2	Tumour	1
Age related macular degeneration	1	Noncompliance	1
Ocular mucous membrane pemphigoid	1	Intellectual disability	1
Previous acanthamoeba keratitis	1	History of herpes zoster	1
Lagophthalmus	1	Atopic dermatitis	1
Endocrine ophthalmopathy	1	Alcoholism	1
Contact lens wearing	1	Systemic lupus erythematosus	1
Fuchs' corneal dystrophy	1	Social isolation in older age	1
Amblyopy	1	Rosacea	1
		Parkinson's disease	1

**Table 4:** Bacteria isolated from cases of infectious keratitis in patients who underwent enucleation or evisceration due to infectious keratitis between January 2007 and December 2018 at the Department of Ophthalmology of Semmelweis University (Budapest, Hungary) (48 eyes of 47 patients).

Bacteria	*n*

*Coagulase negative Staphylococci (CoNS)*	11
*Staphylococcus epidermidis*	7
*Staphylococcus haemolyticus*	1
*Other CoNS*	3
*Staphylococcus aureus*	8
*Streptococcus*	7
*Streptococcus pneumoniae*	3
*Streptococcus pyogenes*	1
*Streptococcus agalactiae*	1
*Other streptococci*	2
*Propionibacterium acnes*	6
*Pseudomonas aeruginosa*	5
*Enterobacter cloacae*	2
*Acinetobacter baumannii*	2
*Capnocytophaga sputigena*	1
*Corynebacterium amycolatum*	1
*Moraxella* sp.	1
*Peptostreptococcus*	1
*Corynebacterium striatum*	1
*Proteus mirabilis*	1

Total	47

**Table 5:** Antibiotic sensitivity for isolated organisms of patients who underwent enucleation or evisceration due to infectious keratitis between January 2007 and December 2018 at the Department of Ophthalmology of Semmelweis University (Budapest, Hungary) (48 eyes of 47 patients).

Bacteria	Antibiotic												
	Amoxicillin	Oxacillin	Cefazolin	Ceftriaxone	Vancomycin	Ciprofloxacin	Ofloxacin	Levofloxacin	Moxifloxacin	Chloramphenicol	Gentamycin	Tobramycin	Neomycin
	% sensitive (number sensitive/number tested)												
*Coagulase negative Staphylococci (CoNS)*	25% (2/8)	45.5% (5/11)	25% (2/8)	n.t.	100% (9/9)	55.6% (5/9)	66.6% (2/3)	37.5% (3/8)	28.6% (2/7)	100% (3/3)	54.5% (6/11)	54.5% (6/11)	100% (3/3)
*Staphylococcus epidermidis*	20% (1/5)	42.9% (3/7)	20% (1/5)	n.t.	100% (5/5)	57.1% (4/7)	100% (1/1)	40% (2/5)	40% (2/5)	100% (2/2)	57.1% (4/7)	57.1% (4/7)	100% (1/1)
*Other CoNS*	33.3% (1/3)	50% (2/4)	33.3% (1/3)	n.t.	100% (4/4)	50% (1/2)	50% (1/2)	33.3% (1/3)	0% (0/2)	100% (1/1)	50% (2/4)	50% (2/4)	100% (2/2)
*Staphylococcus aureus*	83.3% (5/6)	85.7% (6/7)	85.7% (6/7)	n.t.	100% (4/4)	66.7% (4/6)	100% (2/2)	80% (4/5)	83.3% (5/6)	100% (3/3)	100% (7/7)	85.7% (6/7)	100% (2/2)
*Streptococcus*	100% (6/6)	100% (1/1)	n.t.	100% (6/6)	100% (4/4)	n.t.	100% (1/1)	100% (6/6)	100% (5/5)	100% (2/2)	n.t.	n.t.	50% (1/2)
*Streptococcus pneumoniae*	100% (3/3)	100% (1/1)	n.t.	100% (3/3)	100% (2/2)	n.t.		100% (3/3)	100% (3/3)	100% (1/1)	n.t.	n.t.	0% (0/1)
Other *streptococci*	100% (3/3)	n.t.	n.t.	100% (3/3)	100% (2/2)	n.t.	100% (1/1)	100% (3/3)	100% (2/2)	100% (1/1)	n.t.	n.t.	100% (1/1)
*Propionibacterium acnes*	83.3% (5/6)	n.t.	n.t.		n.t.	n.t.	n.t.	n.t.	n.t.	n.t.	n.t.	n.t.	n.t.
*Pseudomonas aeruginosa*		n.t.	n.t.		n.t.	80% (4/5)	n.t.	100% (2/2)	n.t.	n.t.	80% (4/5)	80% (4/5)	n.t.
*Enterobacter cloacae*	0% (0/2)	n.t.	0% (0/1)	100% (2/2)	n.t.	100% (2/2)	n.t.	100% (2/2)	100% (1/1)	n.t.	100% (2/2)	100% (2/2)	n.t.
*Acinetobacter baumannii*	0% (0/1)	n.t.	0% (0/1)	0% (0/1)	n.t.	100% (2/2)	n.t.	100% (2/2)	n.t.	n.t.	100% (2/2)	100% (2/2)	n.t.
*Capnocytophaga sputigena*	100% (1/1)	n.t.	n.t.	100% (1/1)	n.t.	n.t.	n.t.	100% (1/1)	100% (1/1)	n.t.		n.t.	n.t.
*Corynebacterium amycolatum*	n.t.	n.t.	n.t.	n.t.	100% (1/1)	0% (0/1)	n.t.	n.t.	0% (0/1)	n.t.	0% (0/1)	n.t.	n.t.
*Moraxella* sp.	100% (1/1)	n.t.	n.t.	100% (1/1)	100% (1/1)	0% (0/1)	n.t.	100% (1/1)	100% (1/1)	100% (1/1)	n.t.	n.t.	0% (0/1)
*Peptostreptococcus*	100% (1/1)	n.t.	n.t.	n.t.	n.t.	n.t.	n.t.	n.t.	n.t.	n.t.	n.t.	n.t.	n.t.
*Corynebacterium striatum*			n.t.	100% (1/1)	n.t.	100% (1/1)	n.t.	n.t.	n.t.	n.t.	100% (1/1)	n.t.	n.t.
*Proteus mirabilis*	100% (1/1)	n.t.	100% (1/1)	100% (1/1)	n.t.	100% (1/1)	n.t.	100% (1/1)	n.t.	n.t.	100% (1/1)	100% (1/1)	n.t.
% resistant	33.3%	36.8%	50.0%	7.7%	0%	32.1%	16.7%	21.4%	31.8%	0%	23.3%	25%	25%
Total number tested	33	19	18	13	19	28	6	28	22	9	30	28	8

n.t. = not tested.

**Table 6:** Antifungal susceptibility of isolated fungi for patients who underwent enucleation or evisceration due to infectious keratitis between January 2007 and December 2018 at the Department of Ophthalmology of Semmelweis University (Budapest, Hungary) (48 eyes of 47 patients).

Fungi	Antifungal susceptibility			
	Amphotericin B	Fluconazole	Natamycin	Voriconazole
	% sensitive (number sensitive/number tested)			
*Acreconium* sp.	0/1 (0%)	0/1 (0%)	n.t.	1/1 (100%)
*Candida albicans*	1/1 (100%)	1/1 (100%)	n.t.	n.t.
*Candida parapsilosis*	1/1 (100%)	1/1 (100%)	n.t.	1/1 (100%)
*Fusarium*	n.t.	1/1 (100%)	n.t.	1/1 (100%)
*Penicillium* sp.	0/1 (0%)	n.t.	1/1 (100%)	0/1 (0%)
*Trichosporon inkin*	1/1 (100%)	n.t.	n.t.	1/1 (100%)
% resistant	40%	25%	0%	20%

Total number tested	5	4	1	5

n.t. = not tested.

## Data Availability

All data analysed during this study are included in this article.
